# Molecular and serological evidence of systemic *Treponema pallidum* infection in wild western chimpanzees

**DOI:** 10.1016/j.isci.2026.117214

**Published:** 2026-09-03

**Authors:** Clément Labarrere, Hamadou Oumarou Hama, Nicolas Orain, Laia Dotras, Manuel Llana, R. Adriana Hernandez-Aguilar, Florence Fenollar, Oleg Mediannikov

**Affiliations:** 1 IHU-Méditerranée Infection, Marseille, France; 2 Microbes Evolution Phylogeny and Infections (MEPHI), Aix-Marseille Université, AP-HM, Marseille, France; 3 Jane Goodall Institute Spain in Senegal, Dindefelo Biological Station, Dindefelo, Kedougou, Senegal; 4 Department of Social Psychology and Quantitative Psychology, University of Barcelona, Barcelona, Spain; 5 Serra Hunter Programme, Generalitat de Catalunya, Barcelona, Spain; 6 Infectious Risks in the Tropics and Emerging Microorganisms (RITMES), Aix-Marseille Université, SSA, AP-HM, Marseille, France; 7 CNR des Rickettsioses, de la fièvre Q et des bartonelloses, IHU Méditerranée Infection, Marseille, France; 8 IRD, Marseille, France

**Keywords:** Wildlife microbiology, Biological sciences, Zoology

## Abstract

Human treponematoses are chronic diseases caused by closely related *Treponema* species. Increasing evidence of yaws-like *Treponema pallidum* subsp. *pertenue* infections in nonhuman primates raises concerns about pathogen persistence in natural ecosystems. Here, we investigated treponemal infection in western chimpanzees (*Pan troglodytes verus*) using molecular and serological analyses of postmortem skeletal remains from three deceased individuals. Anti-*T. pallidum* antibodies were detected in all chimpanzees using validated serological assays, while molecular assays identified *T. pallidum* DNA in a vertebral bone sample from one individual, supporting systemic dissemination. By integrating forensic findings with retrospective field video observations, we identified concordance between molecular bone involvement and advanced clinical manifestations, including genital lesions and facial depigmentation, in the same chimpanzee. These findings provide rare evidence linking systemic treponemal dissemination to severe disease in a wild endangered great ape and highlight the importance of enhanced surveillance for wildlife conservation and for assessing potential implications for human yaws eradication efforts.

## Highlights

• Anti-*Treponema pallidum* antibodies and DNA were detected in wild chimpanzee remains
• Lab data and field videos reveal advanced systemic treponematosis in chimpanzees
• *Treponema* circulation in apes impacts conservation and yaws eradication

## Graphical abstract

**Figure:**
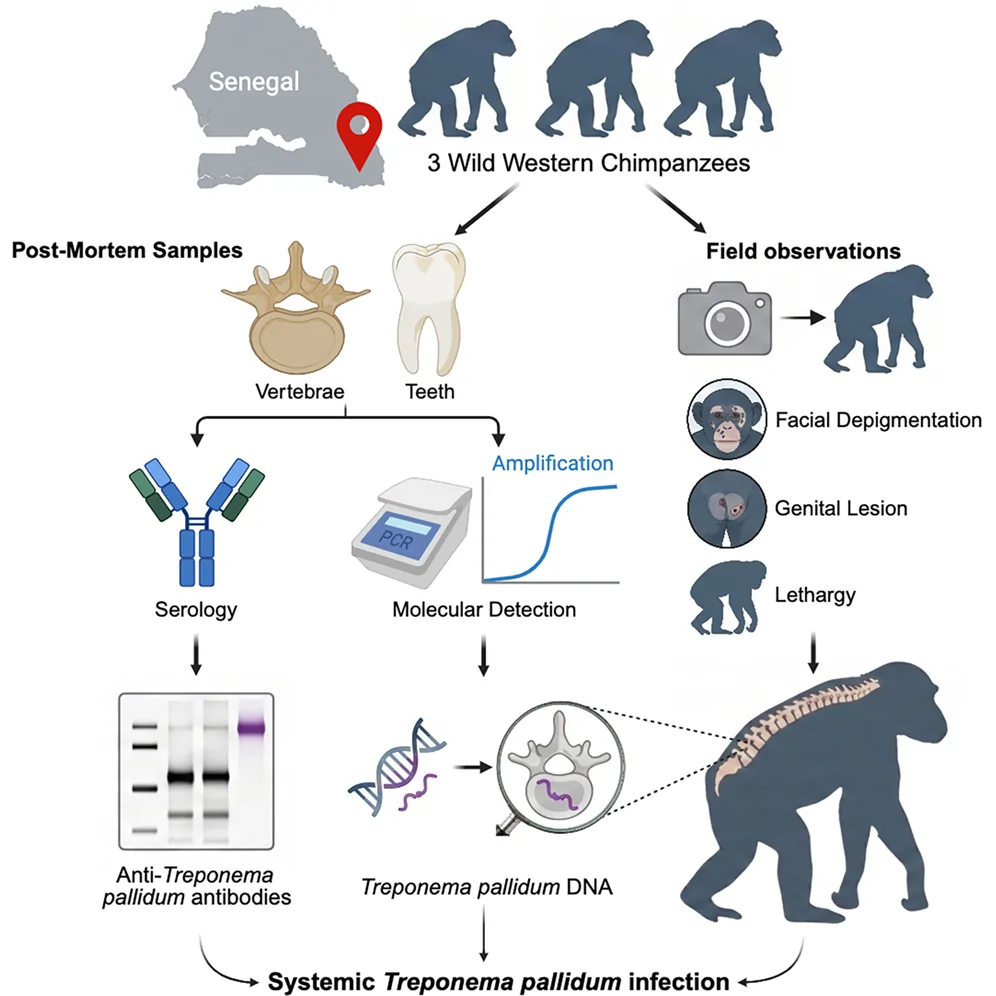

## Introduction

Human treponematoses represent a significant global health concern, imposing a substantial burden of disease. These chronic infections, caused by *Treponema pallidum* subspecies (*pallidum*, *pertenue*, and *endemicum*) and *T. carateum*, are transmitted through sexual or skin contact.^1^ The bacteria preferentially infect mucosal and gelatinous tissues, allowing systemic dissemination.^2^^,^^3^ In the absence of treatment, these infections lead to severe neurological, cardiovascular, visceral, and skeletal complications.^4^^,^^5^^,^^6^ Syphilis, caused by *T. pallidum* ssp. *pallidum*, is the major venereally transmitted human treponematosis, with over 7.1 million adults affected in 2020 (https://www.who.int/fr/news-room/fact-sheets/detail/syphilis). Other endemic treponematoses, including yaws, bejel, and pinta, are classically considered non-venereal infections, although occasional sexual transmission has also been reported for bejel-associated infections.^7^^,^^8^ Global eradication efforts have been launched by the World Health Organization to eliminate yaws (https://www.who.int/Fr/News-Room/Fact-Sheets/Detail/Yaws). However, these eradication efforts face several challenges, including limited treatment distribution, restricted diagnostic resources, and potential nonhuman reservoirs.

Historically, *T. pallidum* was considered anthroponotic, restricted solely to human hosts. This perspective was challenged in 1963 when a strain of *T. pallidum* was isolated from a guinea baboon (*Papio papio*) in Guinea.^9^^,^^10^ This strain, known as Fribourg-Blanc, is antigenically and genetically similar to human yaws and pathogenic to both humans and hamsters (*Mesocricetus auratus*).^11^^,^^12^ For decades, this finding was regarded as anecdotal, as nonhuman primates (NHPs) were rarely investigated for treponemal infections. Nevertheless, recent studies have confirmed that NHPs are also susceptible to treponemal infections, supporting Fribourg-Blanc’s initial findings. For instance, in Senegal, guinea baboons tested positive for treponemal infection through serology.^13^ Similarly, in Tanzania, *T. pallidum* infections were observed in 289 NHPs from eight different species.^14^ These observations highlight the geographical spread of *T. pallidum* subsp. *pertenue* (TPE) infection across Africa and its presence in diverse NHP species, including baboons (*Papio cynocephalus*, *P. papio*, and *P. anubis*), mangabeys (*Cercocebus* sp.), green monkeys (*Chlorocebus sabaeus*), blue monkeys (*Cercopithecus mitis*), patas monkeys (*Erythrocebus patas*), and western red colobus monkeys (*Piliocolobus badius*).^15^^,^^16^

In 2015, an epizootic epidemic of treponematosis in Senegal revealed venereal TPE infections among green monkeys and *P. papio*, with clinical lesions resembling those of human syphilis.^17^ Genetic analyses showed that the strain was nearly identical to Fribourg-Blanc and closely related to TPE, challenging the view of *T. pallidum* as strictly human and highlighting its potential zoonotic epidemiology. A study conducted in 2019 clearly established a link between TPE and yaws-like lesions in a wild western female chimpanzee (*Pan troglodytes verus*).^18^ Additional treponemal infections have been observed in chimpanzees in Cameroon, Uganda, and Ivory Coast and in gorillas (*Gorilla gorilla*) across Cameroon, Gabon, and the Republic of Congo.^15^ Recent findings in Senegal detected specific *T. pallidum* antibodies in 44.8% of fecal samples from western chimpanzees.^19^

These discoveries highlight the need for continued surveillance to assess the impact of yaws-like treponematoses on NHP populations, considering that their health status is affected.^20^ This is particularly critical for great ape species, which are either Endangered or Critically Endangered according to International Union for Conservation of Nature (IUCN).^21^ Furthermore, the circulation of treponemal strains quasi-identical to yaws among NHPs could prove problematic for WHO yaws eradication campaigns.^22^ Although no human treponematosis cases have yet been documented following contact with NHPs, monitoring potential cases remains essential.

This study investigates postmortem samples from western chimpanzees to assess treponemal infection and its potential progression to advanced disease, using molecular and serological analyses of bone specimens.

## Results

### Vertebrae and teeth remains from three deceased chimpanzees were sampled

**Figure 1:**
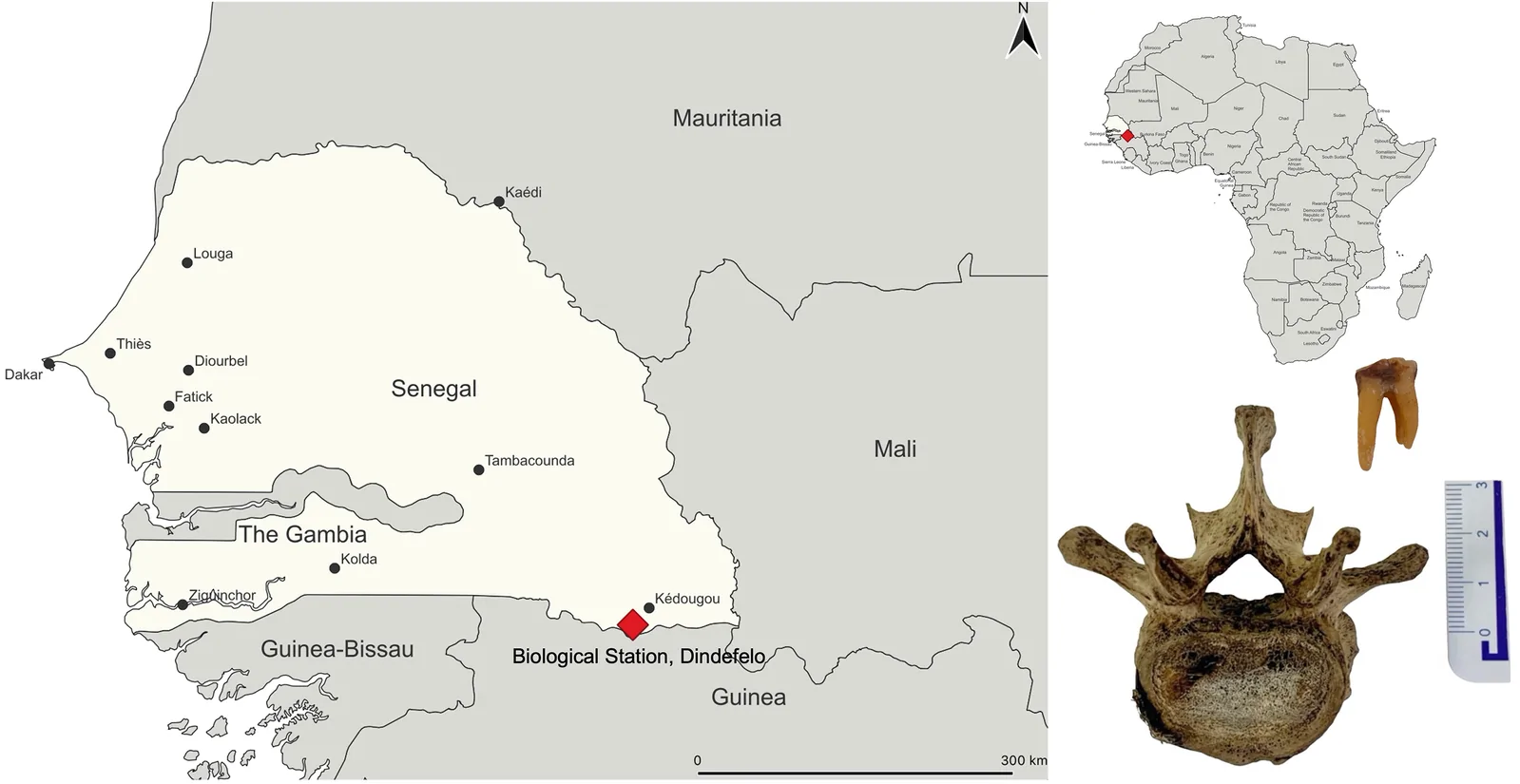
Sampling points for teeth and vertebrae collected in the Dindefelo Community Nature Reserve, Senegal Scale bars, 3 cm.

A total of two vertebrae and four teeth from three chimpanzees were analyzed (Figure 1). A molar, a canine, and a vertebra were obtained from a female chimpanzee who died in May 2016 (female 1), a molar and a vertebra were obtained from a male chimpanzee who died before March 2012 (male 1), and one incisor was obtained from a second male who died in February 2019 (male 2). The samples were obtained from skeletons and thus the soft tissues were no longer present. The cause of the death of these chimpanzees was not identified.

### Detection of *T. pallidum* DNA in the vertebra of a female chimpanzee

**Figure 2:**
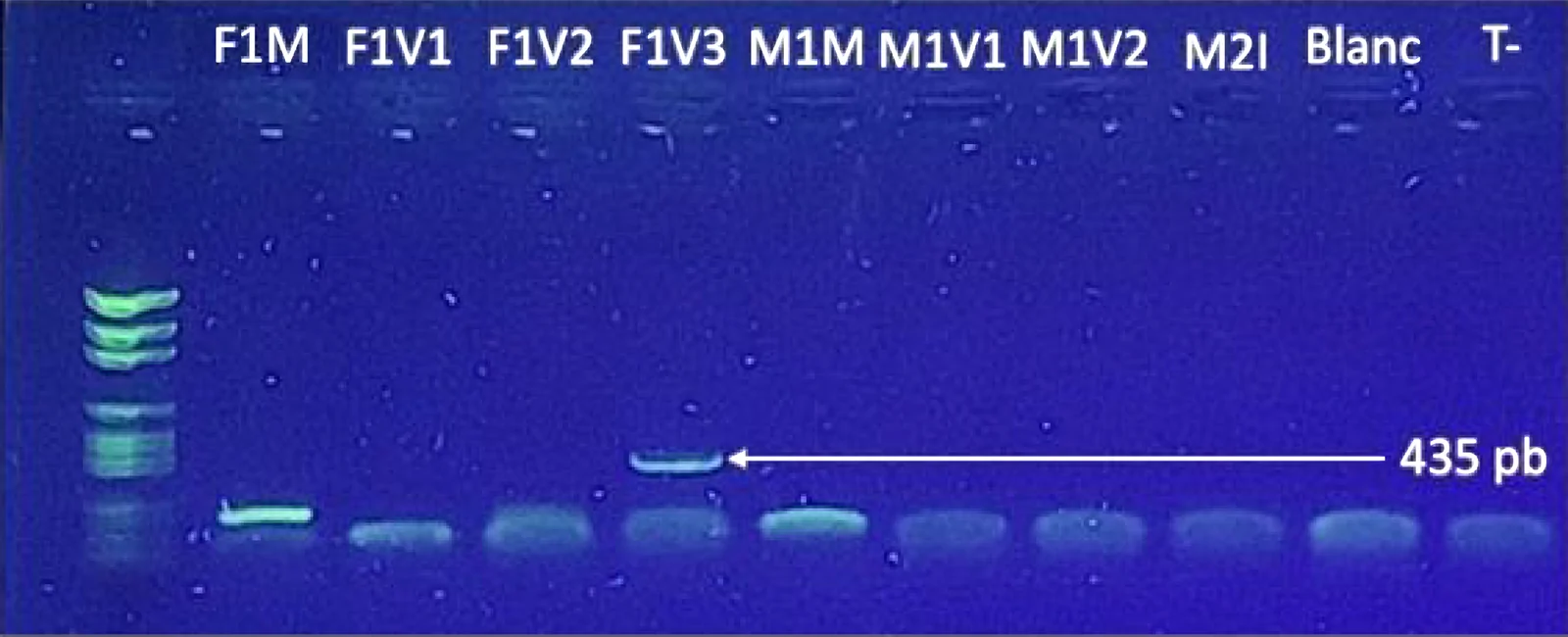
Gel electrophoresis of nested PCR targeting the *TENDBA_0136* gene of the *Treponema pallidum* chromosome, visualized under UV light, from dental pulps and vertebrae of three wild chimpanzees F1M, female 1 dental pulp; F1V, female 1 vertebral body; M1M, male 1 dental pulp; M1V, male 1 vertebral body; M2I, male 2 dental pulp; Blanc, extraction blank; T-, negative PCR control.

The target internal control sequence (TISS) extraction control yielded median Ct values of 31.08 and 32.56, confirming successful DNA extraction. Extracted DNA was screened by qPCR targeting *polA* and *flaA*. Three vertebral DNA extractions from female 1 were positive for *polA* (Ct 30.72 ± 1.56, 31.23 ± 0.80, and 31.98 ± 0.46) and *flaA* (Ct 32.28 ± 1.23, 33.72 ± 1.54, and 34.36 ± 0.48). These Ct values correspond to the mean ± standard deviation (SD) of replicate Ct values obtained from two qPCR runs. All other samples, including female 1’s dental pulp and male 1 and 2 extractions, were negative. Nested PCR targeting the *TENDBA_0136* gene was performed on dental pulp and vertebral DNA (Figure 2). A 435 bp band of moderate intensity was observed in female 1’s vertebra, matching the expected target size. A nonspecific band appeared in the first well. Sequencing attempts performed on the PCR products did not yield interpretable sequences.

### Serological studies

#### Immunoglobulins were recovered from postmortem dental and vertebral samples

Total IgG and IgM assay results are shown in Table S2. Female 1 had median IgG levels of 1,400 pg/mL (interquartile range [IQR]: 800–2,000) in dental pulp and 2,000 pg/mL (IQR: 1,800–6,100) in the vertebral body. Male 1 showed 3,700 pg/mL in dental pulp and 2,750 pg/mL (IQR: 1,400–8,500) in the vertebral body. IgG levels in male 2 were zero. Median IgM levels were 11.7 pg/mL (IQR: 9.3–15.9) for female 1, 11.2 pg/mL (IQR: 8.5–15) for male 1, and zero for male 2.

#### Recombinant and native treponemal antigens showed expected protein profiles

The SDS-PAGE electrophoresis gels are shown in Figure S1. A band slightly above 72 kDa corresponds to the recombinant treponemal proteins (p15, p17, and p47) as indicated by the manufacturer. Another band is observed at 35 kDa; this can be explained by a purity rate that is not 100%. The native antigen electrophoresis gel shows several bands between 250 and 17 kDa, consistent with previous observations.^23^

#### Anti-*T. pallidum* antibodies were detected in all three chimpanzees by automated western blot

First, western blot migration standards were analyzed. Subsequently, the signal-to-noise ratio (S/N) was assessed for each sample at the expected molecular weight (MW) to determine the presence or absence of anti-*T. pallidum* antibodies. Antibodies classified as positive for anti-*T. pallidum* exhibited an S/N significantly higher than that of the median obtained from a series of syphilis-negative sera (*n* = 10). The positive human sera exhibited a distinct band at 70 kDa. The 70 kDa signal observed with the automated western blot system is consistent with the expected 77 kDa recombinant antigen, slight differences being attributable to migration variability between conventional SDS-PAGE and capillary-based electrophoresis systems.

**Figure 3:**
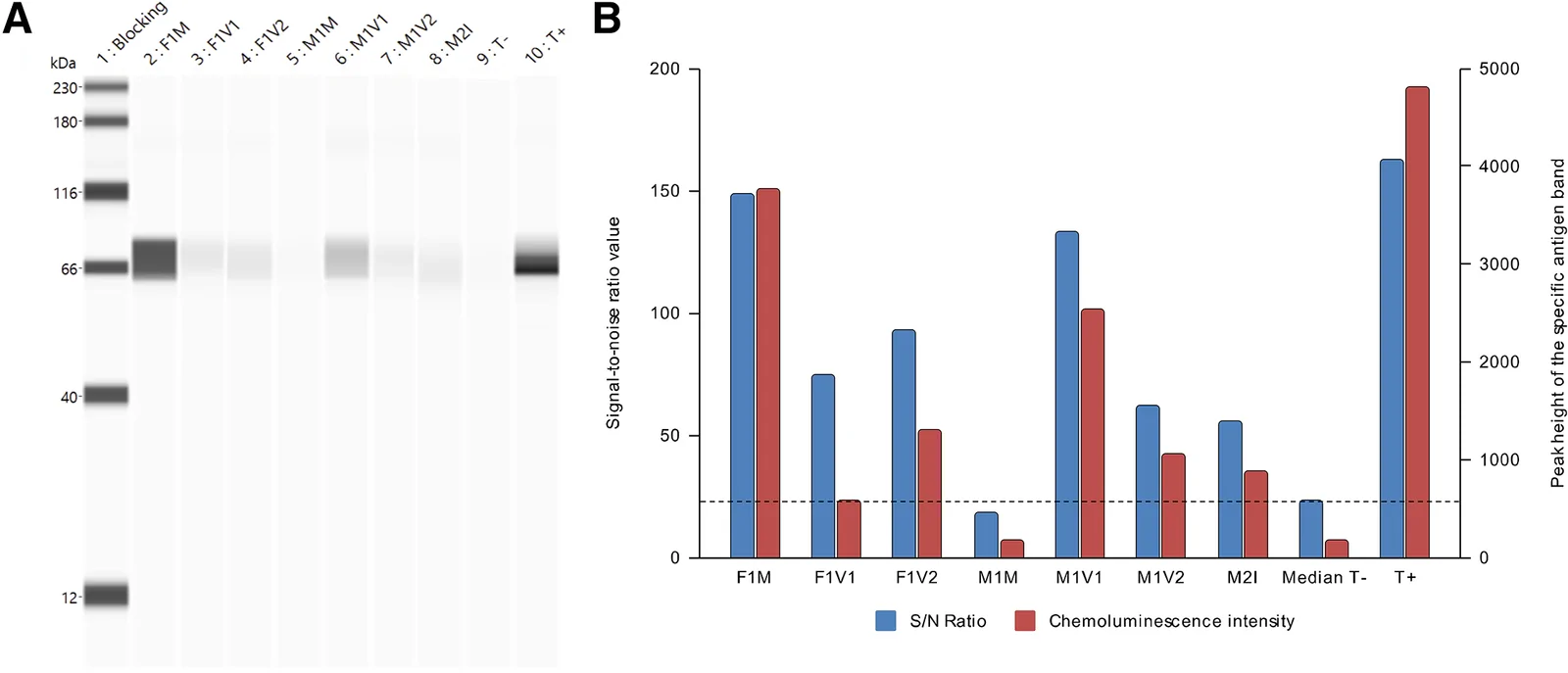
Serological detection of *Treponema pallidum* antibodies in chimpanzee tissue samples using western blot analysis (A) Western blot migration of recombinant *Treponema pallidum* antigen with extracted antibodies from dental pulp and vertebrae, showing a single band per capillary at the expected molecular weight (70 kDa). (B) Signal-to-noise ratio (S/N) and chemiluminescence intensity of the same samples as reported by Compass software without data modification; the dotted line represents the median S/N ratio of syphilis-negative sera (*n* = 10). F1M, female 1 dental pulp; F1V1 and F1V2, female 1 vertebral body replicates; M1M, male 1 dental pulp; M1V1 and M1V2, male 1 vertebral body replicates; M2I, male 2 dental pulp; median T-, median of negative controls; T+, positive control. Data points in the bar chart represent single, individual measurements per sample/replicate; no pooled data or error bars are included.

Migration standards for tests with the recombinant antigen showed perfect migration (Figure 3). Negative controls displayed no bands (S/N median = 23.25, IQR: 10.9–31.7), while positive controls showed a single 70 kDa band, corresponding to the expected MW. Bands of the same 70 kDa were detected in female 1’s dental pulp (S/N = 148.8) and vertebra (S/N = 83.7, IQR: 74.5–92.9), male 1’s vertebra (S/N = 97.45, IQR: 62.1–132.8), and male 2’s dental pulp (S/N = 55.9). These data indicate the presence of anti-*T. pallidum* antibodies in female 1 (dental pulp and vertebral body), male 1 (vertebral body), and male 2 (dental pulp). For western blot with the native *T. pallidum* antigen, sample migration in the capillaries was unsuccessful. Despite multiple tests with native antigen and positive syphilis sera, results were inconclusive, preventing testing of the extracted antibodies with Jess western blot.

#### Anti-*T. pallidum* antibodies were confirmed in postmortem samples by mini line blot

**Figure 4:**
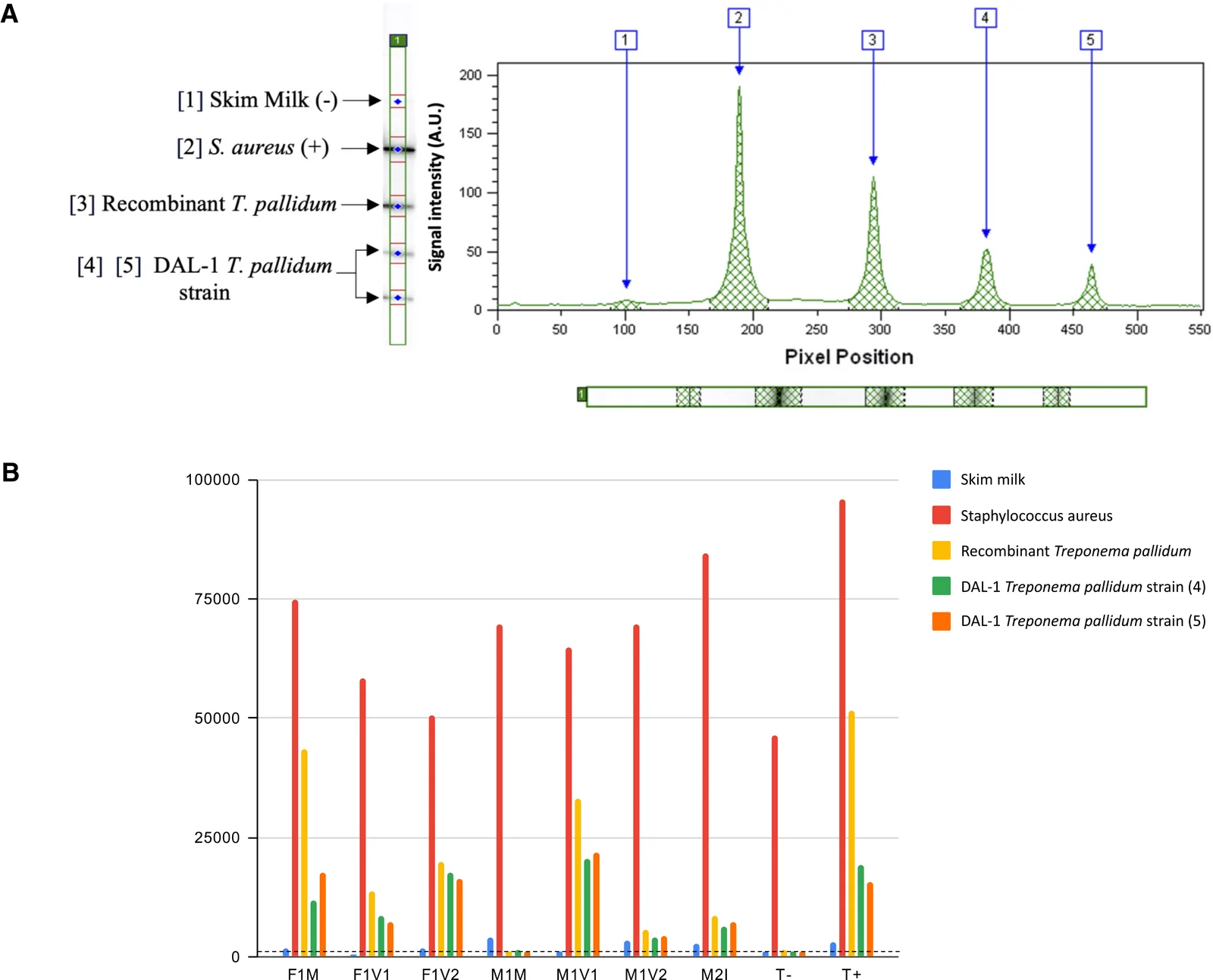
Evaluation of antibody reactivity to *Treponema pallidum* antigens using mini line blot assays (A) Representative mini line blot assays performed on the dental pulp from female 1, illustrating antibody reactivity to *Treponema pallidum* antigens. The quantification was performed using the FUSION FX chemiluminescent imaging system and ImageQuantTL software (Witec AG, Sursee, Switzerland); the *x* axis shows pixel coordinates as proxies for antigen positions, and the *y* axis represents serological signal intensity in arbitrary units (a.u.). (B) Quantified antibody responses for each antigen tested on the samples from the three chimpanzees, with a dotted line indicating the cutoff set at 2,000 a.u. F1M, female 1 dental pulp; F1V1 and F1V2, female 1 vertebral body replicates; M1M, male 1 dental pulp; M1V1 and M1V2, male 1 vertebral body replicates; M2I, male 2 dental pulp; T-, negative controls; and T+, positive control. All bars represent individual quantitative signal intensity values for each specific sample or replicate; no statistical pooling or error bars are applicable.

The results of the migration of the extracted antibodies in the presence of recombinant antigen and native *T. pallidum* antigen are presented in Figure 4. No fluorescence was observed in negative controls (skimmed milk and negative syphilis serum), while the *S. aureus* positive control fluoresced on all strips. Extracted antibodies from female 1 (dental pulp and vertebral body), male 1 (vertebral body), and male 2 (dental pulp) reacted specifically with both antigens, confirming the automated western blot results and the presence of anti-*T. pallidum* antibodies in these samples.

### Combined molecular, serological, and field data support advanced treponemal infection in female 1

In conclusion, female 1 presented a positive vertebral sample for *T. pallidum* in molecular analysis, along with the positive serological results in both the vertebra and dental pulp. While establishing the definitive, proximal cause of death remains a classic challenge in wildlife forensics, the convergence of molecular, serological, and antemortem behavioral data strongly implies that this individual suffered from a severe, debilitating stage of advanced treponematosis at the time of death. In contrast, only serology yielded positive results for male 1 and male 2. Male 1 tested positive in both tooth and vertebra, while male 2 was positive in tooth only, confirming *T. pallidum* infection; however, definitive conclusions regarding the cause of death for these individuals remain uncertain (Table 1).

**Table 1:** Probable *Treponema pallidum* infection status of three chimpanzees based on molecular and serological results

Individual	Sample	*T. pallidum* DNA detection	Immunoassay	Western blot	Mini line blot	Probable infectious status
Female 1	teeth	negative	high IgG and low IgM	positive	positive	advanced stage of treponemal infection
	vertebra	positive				
Male 1	tooth	negative	high IgG and low IgM	positive	positive	treponemal infection
	vertebra					
Male 2	tooth	negative	IgG and IgM negative	positive	positive	ambiguous: conflicting antibody results

## Discussion

We should note that all samples were forensic and came from skeletons recovered several years ago, and in one case more than a decade ago, and stored at ambient temperature in the field. Poor preservation likely affected the results due to nucleic acid and protein degradation and environmental exposure.

Our findings confirm that apes are susceptible to treponemal infections, in line with previous research. Serological analyses identified anti-*T. pallidum* antibodies in all three individuals. The presence of these antibodies was observed by western blot and mini line blot in both the dental pulp and vertebral bodies of female 1 and male 1, as well as in the dental pulp of male 2. This strongly indicates that every individual had been infected with *T. pallidum* during their lifetime.

Total immunoglobulin assays showed high IgG levels associated with comparatively low IgM levels in female 1 and male 1, supporting previous or long-standing exposure to *T. pallidum* rather than an acute primary infection. The detection of IgM antibodies in a subset of infected individuals may reflect the persistence of low-level or fluctuating IgM responses during chronic treponemal infection, as described in other human spirochetal diseases.^24^^,^^25^ In male 2, only anti-*T. pallidum* antibodies were detected in the dental pulp, contrasting with negative IgG and IgM results. This discrepancy likely reflects the extremely limited and heterogeneous nature of the dental pulp sample rather than a methodological error. Our antibody-based approach offers the advantage of not being susceptible to contamination or false positives, though IgM and IgG specificity does not allow identification of the treponemal subspecies.

**Figure 5:**
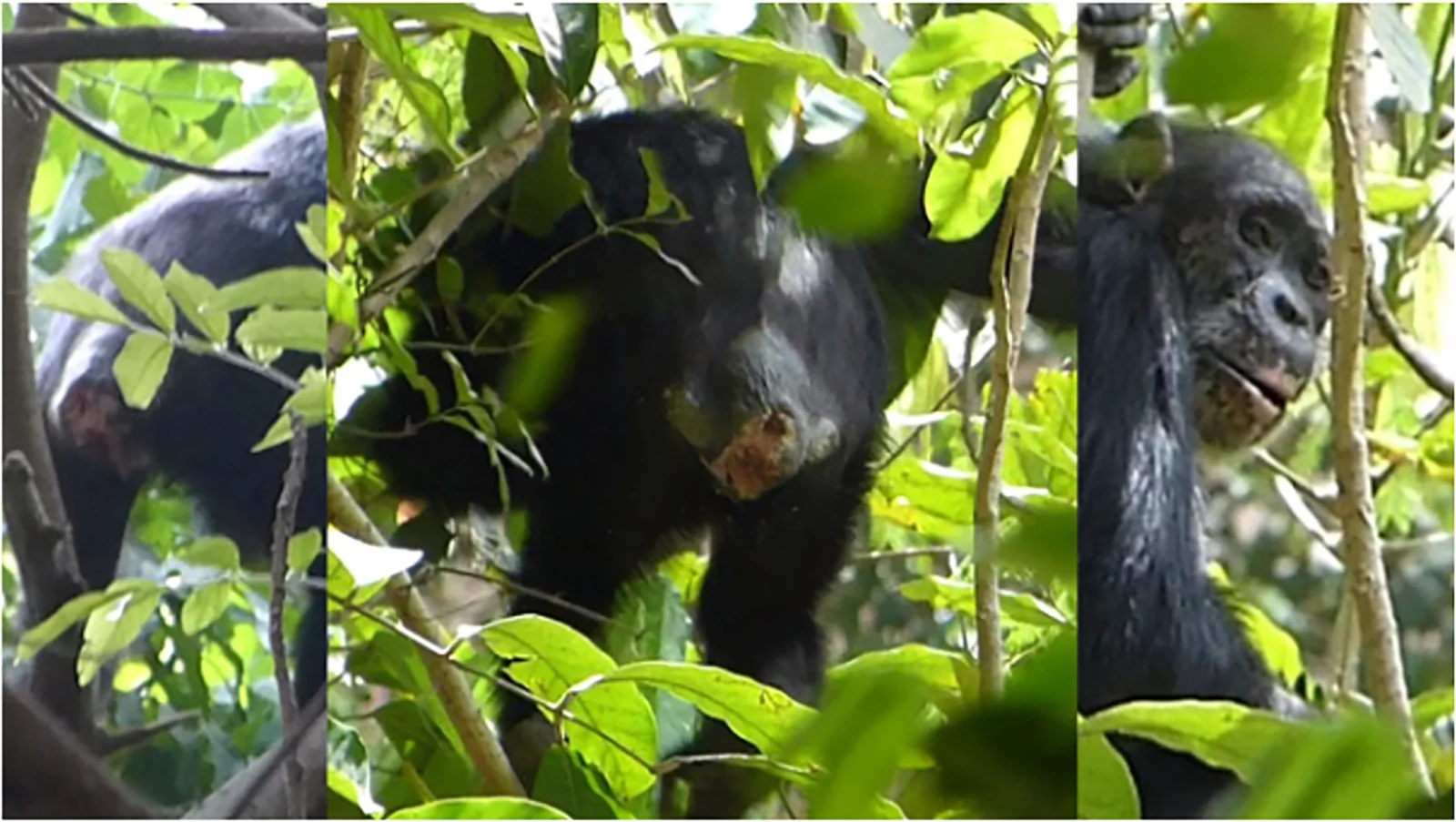
Female 1 at the Dindefelo Community Nature Reserve on 25 April 2015 Lesions that appear consistent with a treponemal infection are visible in her genital area. Her face shows clearly marked treponemal depigmentation. Still images were extracted from video footage obtained opportunistically by the Jane Goodall Institute Spain in Senegal.

Molecular analysis detected *T. pallidum* in the vertebral bone marrow of female 1, consistent with skeletal involvement associated with advanced treponemal infection.^1^ The presence of treponemal DNA in bone, together with antibodies in dental pulp and bone marrow, suggests that female 1 was experiencing a late, systemic stage of infection. While we cannot definitively rule out alternative co-morbidities as the immediate cause of death, such advanced skeletal involvement underscores the fatal potential of unmanaged treponematosis in wild great apes. Although syphilitic gummata commonly develop in spongy bone tissue during tertiary stages, no macroscopic lesions were observed upon examination of this old vertebra.^1^ Finally, the absence of *T. pallidum* DNA in the dental pulp aligns with the known biology of treponemal infection and indicates that there was likely no bacteremia at the time of death, as treponemes typically circulate only during early stages.^1^ Discovered *post-factum* field observations support the conclusion that female 1 suffered from an advanced treponemal infection. Opportunistic video footage from the Jane Goodall Institute Spain in Senegal (April and October 2015) shows female 1 frequently touching and smelling her fingers after contacting her genital area, which appeared red and lesioned, consistent with treponemal infection (Figure 5). Facial depigmentation, also observed, is consistent with advanced-stage treponemal lesions.^26^ The female appeared to be tired and rested frequently. The concordance between advanced skeletal infection and observed behavioral and clinical signs (genital contact, lethargy, and facial depigmentation) supports the integration of postmortem molecular evidence, serological data, and retrospective behavioral observations. Together, these approaches provide a comprehensive framework for interpreting systemic treponemal infection and its multisystem clinical manifestations in wild great apes.

Contextualizing these findings, a recent study reported anti-*T. pallidum* antibodies in 44.8% of western chimpanzees from the same reserve, indicating active circulation of *T. pallidum* in this population.^19^ This hypothesis is further supported by the presence of an individual from the present study who died in 2016 and showed signs of advanced infection, a stage known to occur several years post-inoculation. Overall, these data suggest that *T. pallidum* circulation in this reserve may date back more than a decade, which is consistent with the detection of anti-*T. pallidum* antibodies in an individual who died before March 2012.

Moreover, in 2016, an emerging epizootic of venereal TPE was reported in green monkeys at the Niokolo-Koba National Park study site in Senegal and spread to guinea baboons the following year.^17^ The genome analysis conducted in that study revealed an accelerated mutation rate, suggesting significant dissemination. This clone may also have infected the chimpanzees in our study. The green monkey group studied in 2016 lived about 88 km from our current study site, and lesions resembling human syphilis were reported, highlighting the potential for cross-species transmission of treponemal infections among NHPs. Finally, though the analyzed bones were not directly linked to field diagnoses, previous reports have described significant physical impairment in infected chimpanzees, demonstrating the severe impact of treponemal infections on both physical health and survival. These infections, progressing to advanced stages, may ultimately be fatal, as evidenced by the likely cause of death in female 1 in our study.^18^

With an estimated population of only 53,000 individuals, West African chimpanzees are classified as Critically Endangered on the IUCN Red List.^27^ The threat of treponematoses thus represents a serious risk to both the health and survival of chimpanzees. Additionally, treponematoses could significantly impact chimpanzee social behavior and reproduction, as observed in gorillas^20^ and olive baboons (*P. anubis*).^28^ This highlights an urgent need to increase health surveillance for NHPs, not only to protect these animals but also to aid in their conservation.

Moreover, controlling the spread of this pathogen is essential for understanding the potential transmission risks to human populations, as insufficient monitoring could allow the emergence of a highly transmissible epidemic clone. A critical concern remains whether NHPs could serve as a reservoir for yaws-like TPE infections in humans. In West Africa, treponematoses in NHPs are caused by the Fribourg-Blanc strain, which is nearly genetically identical to the yaws-causing strain and is pathogenic to humans.^9^^,^^10^^,^^11^ Its presence in NHP populations could therefore complicate yaws eradication efforts.

### Limitations of the study

The extremely limited amount of available biological material, particularly dental pulp and bone extracts, restricted the possibility of repeated analyses and the application of complementary assays, including more extensive sequencing approaches that could have further refined pathogen characterization. Serological assays used in this study were originally developed for human immunoglobulins, and species-specific validation in chimpanzees was not possible due to the lack of reference sera from experimentally infected individuals. Finally, no macroscopic skeletal lesions consistent with tertiary treponematosis were observed in the vertebral specimen, likely due to the advanced degradation state of the bone material and the limitations inherent in postmortem preservation.

## Resource availability

### Lead contact

Requests for further information and resources should be directed to and will be fulfilled by the lead contact, Oleg Mediannikov (olegusss1@gmail.com).

### Materials availability

This study did not generate new unique reagents.

### Data and code availability

• Data reported in this paper will be shared by the lead contact upon request.
• This paper does not report original code.
• Any additional information required to reanalyze the data reported in this paper is available from the lead contact upon request.

## Acknowledgments

We sincerely thank Masse Sambou and Sacy Nadaradjane for their valuable help with the material logistics necessary for this work. We are grateful to the Direction des Eaux, Forêts, Chasses et de la Conservation des Sols (Senegal) for granting permission to collect the chimpanzee samples analyzed in this study. We thank the field assistants and volunteers from the Jane Goodall Institute Spain in Senegal for their help with sample collection in the Dindefelo Community Nature Reserve (Senegal). This work was supported by the Institut Hospitalo-Universitaire (IHU) Méditerranée Infection, the French National Research Agency (ANR) under the “Investissements d’avenir” program (reference ANR-10-IAHU-03), the 10.13039/501100008530European Regional Development Fund (FEDER PRIMI), and the Région Provence-Alpes-Côte d’Azur.

## Author contributions

Conceptualization, C.L., H.O.H., F.F., and O.M.; methodology, C.L., H.O.H., F.F., and O.M.; software, C.L., H.O.H., and N.O.; formal analysis, C.L., H.O.H., N.O., and O.M.; investigation, C.L., H.O.H., L.D., M.L., R.A.H.-A., F.F., and O.M.; resources, C.L., L.D., M.L., R.A.H.-A., F.F., and O.M.; data curation, C.L., H.O.H., N.O., and O.M.; writing – original draft, C.L. and O.M.; writing – review and editing, all authors; supervision, F.F. and O.M.; funding acquisition, F.F. and O.M.

## Declaration of interests

The authors declare no competing interests.

## STAR★Methods

### Key resources table

**Table:** 

REAGENT or RESOURCE	SOURCE	IDENTIFIER
**Antibodies**		

Goat anti-human IgG, IgA and IgM antibody conjugated to HRP	Jackson ImmunoResearch, UK	Cat# 109-035-064; RRID: AB_2337583

**Biological samples**		

Chimpanzee postmortem skeletal remains (vertebrae and teeth), collected in this study	Jane Goodall Institute Spain skeletal collection, Senegal	Female 1, Male 1, Male 2
Human serum samples	This study	N/A

**Chemicals, peptides, and recombinant proteins**		

Recombinant *T. pallidum* fusion antigen (p15, p17, p47)	Antibodies-Online GmbH, Germany	Cat# ABIN934736
Native *T. pallidum* strain DAL-1 crude antigen	This study	N/A

**Critical commercial assays**		

Human IgM ELISA Kit	Abcam, UK	Cat# ab214568
Human IgG ELISA Kit	Abcam, UK	Cat# ab100547
Qiagen MinElute Silica Spin column	Qiagen, Germany	Cat# 28004
Qubit HS Assay Kit	Thermo Fisher Scientific, USA	Cat# Q32851
LightCycler 480 Probes Master	Roche, Switzerland	Cat# 04707494001
BigDye Terminator Cycle Sequencing Kit	Thermo Fisher Scientific, USA	Cat# 4337455
NucleoFast 96 PCR Plate	Macherey-Nagel, France	Cat# 743100.10
Anti-*Treponema pallidum* Western Blot (IgG/IgM)	Euroimmun, Germany	Cat# DY 2111-1601 G (IgG)/DY 2111-1601 M (IgM)
Jess 12–230 kDa Separation Module	ProteinSimple, USA	Cat# SM-W004

**Oligonucleotides**		

Target Internal Control Sequence (TISS)	Sow et al.^29^	N/A
See Table S1 for sequences of primers and probes used for the detection of *Treponema pallidum*	This study	N/A

**Software and algorithms**		

CFX Maestro Software	Bio-Rad Laboratories, USA	
Compass for SW software	ProteinSimple, USA	Version 6.3
ImageQuantTL	Witec, Switzerland	N/A

**Other**		

2 kDa MWCO Dialysis Cassettes	Thermo Fisher Scientific, USA	Cat# 69580

### Method details

#### Ethical statement

Chimpanzee samples were obtained from the Jane Goodall Institute Spain in Senegal skeletal collection housed at its Biological Station in Dindefelo. The skeletons were recovered after the burial of individual chimpanzees found dead in the field. Transport of the samples for this study was granted by the Ministry of Environment and Ecological Transition of Senegal (permit 0068/IREF/KDG from the Direction des Eaux, Forêts, Chasses et de la Conservation des Sols, April 8th 2025).

Human serum samples used in this study consisted of residual serum remaining after routine diagnostic testing and collected during routine clinical care at the infectious diseases diagnostic laboratory of Marseille University Hospitals (AP-HM, Marseille, France). The study exclusively involved the secondary use of fully anonymized residual biological material and did not include any additional sampling or intervention involving patients. In accordance with Articles L.1121-1 et seq. of the French Public Health Code governing research involving human participants (“Loi Jardé”), studies based solely on the reuse of anonymized residual biological samples collected during routine care do not require specific ethics committee or Institutional Review Board approval in France. Patients are informed that residual samples may be reused for research purposes and may object through the hospital’s Data Protection Officer. None of the patients whose residual sera were used in this study objected to such reuse.

#### Sample collection

The samples analyzed come from the Dindefelo Community Nature Reserve in southeastern Senegal. Human sera served as positive and negative controls for validation of the serological assays.

#### Sample preparation and inspection

A visual inspection was performed to identify macroscopic lesions. External surfaces of the samples were disinfected with ethanol and bleach. Dental pulp was recovered by fracturing teeth and using a sterile probe in a low-bind Eppendorf tube, and vertebral bone marrow was collected similarly.

#### Molecular biology

All protocols were executed under a hood with a filtered negative air pressure and dedicated only to this type of samples (Paleomicrobiology and Paleoserology). During analyses, no simultaneous addition of positive control with the samples was carried out. DNA extraction was performed according to the method previously described.^30^ Briefly, dental pulp was mechanically powdered using a sterile dental scaler. For each sample, approximatively 30 mg of material was resuspended in 1 mL of lysis buffer (0.45 M EDTA, pH 8.0 and 0.25 mg/mL proteinase K) and spiked with 1 μL of a Target Internal Control Sequence (TISS, an exogenous synthetic DNA) to monitor extraction efficiency.^29^ After a 15h incubation at 37 °C on a thermomixer (500 rpm), the DNA was captured onto a silica membrane using a high-salt guanidine hydrochloride binding buffer and purified via a Qiagen MinElute Silica Spin column. The purified DNA was subsequently eluted in 25 μL of nuclease-free water. For quality control, total DNA was quantified using the Qubit HS assay.

All DNA samples were screened by quantitative PCR (qPCR) targeting *T. pallidum polA* and *flaA* genes (Table S1) on a CFX Opus Real-Time PCR System (Bio-Rad Laboratories, Hercules, United States).^31^^,^^32^ Reactions contained 5 μL DNA, 10 μL LightCycler 480 Probes Master (Roche, Bâles, Switzerland), 3.5 μL DNase/RNase-free water, and 0.5 μL of probe (5 μM) and 0.75 μL of primers (20 μM). Amplification was 95°C for 10 min, followed by 40 cycles of 95°C for 10 s and 60°C for 30 s.

Extracted DNA was amplified by nested PCR targeting a *T. pallidum* chromosomal gene (*TENDBA_0136*) as previously described (Table S1).^17^ PCR products were purified using Macherey-Nagel plates (Macherey-Nagel EURL, Hoerdt, France) and sequenced with the BigDye Terminator Cycle sequencing kit (Thermo Fisher Scientific) on an automated sequencer (Applied Biosystems 3500 Genetic Analyzer, Thermo Fisher Scientific). The sequencing program was 95°C for 15 min, followed by 25 cycles of 95°C for 30 s, 50°C for 5 s, and 60°C for 3 min.

#### Protein extraction

Protein extraction was performed as previously described.^33^ Briefly, samples were incubated in 200 μL of ethylenediaminetetraacetic acid (EDTA) and subjected to sonication (5 × 1 min, 6,000 J), followed by overnight incubation at 4°C under agitation. After centrifugation (30 min, 17,000g, room temperature), the supernatant (EDTA fraction) was collected and stored at −20°C. The remaining pellet was washed twice with sterile distilled water, resuspended in 100 μL of 50 mM ammonium bicarbonate (pH 7.4), and incubated for 48 h at 75°C. After centrifugation (30 min, 17,000g), the supernatant (bicarbonate fraction) was collected and stored at −20°C. EDTA and bicarbonate fractions were then pooled and dialyzed twice using 2 kDa MWCO dialysis cassettes against 50 mM ammonium bicarbonate (4 h, then overnight). The dialyzed extract was freeze-dried for 24 h at −80°C, and the resulting lyophilized material was resuspended in 30 μL of 1X DPBS (Thermo Fisher Scientific) and stored at −20°C until analysis.

#### Quantification of total IgG and IgM and specific IgA, IgG, and IgM of T. pallidum

##### ELISA

Total IgM and IgG were measured by ELISA using immunoassay kits (Abcam, Cambridge, UK). The Human IgM ELISA kit (ab214568) and the Human IgG ELISA kit (ab100547) were performed according to the manufacturer’s protocols. Although the ELISA kits were designed for human immunoglobulins, their use in chimpanzee samples was considered appropriate due to the high conservation of IgG and IgM structures among primates and the expected cross-reactivity of assay components with chimpanzee immunoglobulins.

#### Automated western blot

The Jess™ Simple Western system (ProteinSimple, San Jose, USA) is an automated system for capillary-based size separation and nano-immunoassays. Two *T. pallidum* antigens were used. The first consist of a commercial recombinant antigen (ABIN934736) (Antibodies-Online GmbH, Aachen, Germany) composed of a fusion of three major proteins of *T. pallidum* (p15, p17, p47), with a molecular weight (MW) of 77 kDa.^23^^,^^34^ The second consisted of a crude antigenic preparation derived from *T. pallidum* strain DAL-1 obtained from rabbit testicular tissue experimentally infected with *T. pallidum*. The DAL-1 antigen was selected because major immunogenic proteins of *T. pallidum* are highly conserved across subspecies, including yaws-causing strains, allowing reliable serological cross-reactivity for antibody detection.^35^ Bacteria were purified by Percoll density gradient centrifugation, followed by washing and sonication to produce the antigenic extract.^19^ This crude extract was used to detect anti-*T. pallidum* antibodies through the binding of sample antibodies to immobilized treponemal proteins. An SDS-PAGE electrophoresis was performed for both proteins using a 12% polyacrylamide gel.

To evaluate anti-treponema IgA, IgM, and IgG, a Western blot was performed using the Jess™ Simple Western System (ProteinSimple) and the Jess™ 12–230 kDa separation module (SM-W004, ProteinSimple). The protocol used corresponds to that of the manufacturer.^36^ Western blot was carried out with the proteins of the strain DAL-1 (0.8 μg/μL) and the recombinant protein (0.5 μg/μL). A goat anti-human IgG, IgA and IgM antibody conjugated to HRP (1:500) (Immunology Jackson ImmunoResearch, Ely, UK) was used to reveal binding of primary antibodies to antigens and chemiluminescent revelations were established with peroxide/luminol-S (ProteinSimple). Anti-human secondary antibodies were used, as cross-reactivity with nonhuman primate immunoglobulins, including chimpanzee IgG and IgM, is expected due to the high conservation of immunoglobulin structures among primates. The performance of this assay was validated using human sera previously confirmed as positive for anti-*T. pallidum* antibodies by a commercial Western blot (Euroimmun, Lübeck, Germany), as well as negative control sera. Two positive controls (1:5000 dilution of syphilis serum) and two negative controls were included.

#### Mini line blot

A Mini Line Blot was prepared by incorporating the native antigen and the recombinant antigen of *T. pallidum*. *Staphylococcus aureus* served as a positive control (non-specifically binding human immunoglobulins^33^) and negative controls were added: skimmed milk and negative syphilis sera.^37^ This technique was carried out as previously described.^33^ 1 μL of serum as well as 15 μL of extracted antibodies, diluted in PBST-M 0.5% to 1 mL, were added to the wells. Anti-human IgG, IgA and IgM antibodies conjugated to horseradish peroxidase (1:1000 in 0.5% PBST-M) and 1 mL of peroxide/luminol (1:1) were added. To avoid cross-contamination with positive sera, extracted antibody samples were tested separately.

### Quantification and statistical analysis

For serological signal quantification and validation, absorbance values obtained from ELISA kits were quantified against standard curves according to the manufacturer’s instructions, with concentration values presented as medians with interquartile ranges (IQR). Automated Western blot digital images were analyzed using Compass for SW software (ProteinSimple), and the quantified data of detected proteins were reported as chemiluminescence intensity and signal-to-noise ratio. Finally, for the Mini Line Blot assay, strips were imaged with a Fusion FX system (VILBER, Marne La Vallée, France), and quantification was performed using ImageQuantTL software (Witec, Sursee, Switzerland) with chemiluminescent signal intensity measured in arbitrary units (A.U.) and a strict positivity cutoff threshold set at 2,000 A.U. All measurements plotted in bar graphs (Figures 3B and 4B) represent discrete, single individual sample values or single extraction replicates. No data were statistically pooled across different individuals, and no calculation of sample means or standard error bars was applicable for these distinct observations.
