# Supplementary material for: Molecular and serological evidence of systemic Treponema pallidum infection in wild western chimpanzees
Source: iScience. 2026 Sep 3;29(9):117214. doi: 10.1016/j.isci.2026.117214 (PMC13571651; doi:10.1016/j.isci.2026.117214)

**iScience, Volume *29***

## Supplemental information

# Molecular and serological evidence of systemic *Treponema pallidum* infection in wild western chimpanzees

**Clément Labarrere, Hamadou Oumarou Hama, Nicolas Orain, Laia Dotras, Manuel Llana, R. Adriana Hernandez-Aguilar, Florence Fenollar, and Oleg Mediannikov**

Table S1. Sequences of primers and probes used for the detection and identification of *Treponema pallidum* subspecies

| PCR | Gene targeted | Name | Primers (5'-3') and probes | Temperatures of fusion (°C) | Amplicons (pb) | References |
|---|---|---|---|---|---|---|
| qPCR | *polA* (DNA polymerase I) | polA.F | GTCGAGACTGA AAAGGAGTGCA | 60 | 122 | (*29*) |
| | | polA.R | GTGAGCGTCTC ATCATTCCAAAG | | | |
| | | polA.P | 6FAM-TGCTGTGCAGGATCC GGCATATGTCC | | | 15/07/2026 11:02:00 |
| qPCR | *flaA* (flagellar filament outer layer protein) | flaA.F | GCGGTTGCACA GTGGGAG | 60 | 61 | |
| | | flaA.R | CAGCATGGGCG ACAGGAT | | | |
| | | flaA.P | 6FAM-TTGTGCTGAA TTCTTCCGC GG | | | |
| PCR | *TENDBA_0136* strain Strasbourg_6715 | F1_TENDBA _0136 | TGCATTCACATC TGGCTCGT | 60 | 633 | (*20*) |
| PCR | *TENDBA_0136* strain Strasbourg_6715 | R1_TENDBA _0136 | GCAATGTGCAC AAGAGTCCG | | | |
| Nested PCR | *TENDBA_0136* strain Strasbourg_6715 | F2_TENDBA _0136 | CAGCTCGTGCA TTCACATTA | 55 | 435 | (*20*) |
| Nested PCR | *TENDBA_0136* strain Strasbourg_6715 | R2_TENDBA _0136 | ACACAACGGCT GCGAATACT | | | |

Table S2. Concentrations of total immunoglobulins IgG and IgM obtained by ELISA assay from dental pulps and vertebrae. IgG and IgM concentrations were reported as medians with interquartile ranges (IQR).

| Sex | Date of death | Sample | Median IQR IgG (pg/mL) | Median IQR IgM (pg/mL) |
|---|---|---|---|---|
| Female 1 | May 2016 | Dental pulp | 1,400 (800–2,000) | 0 |
| | | Vertebra | 2,000 (1,800–6,100) | 11.7 (9.3–15.9) |
| Male 1 | Before March 2012 | Dental pulp | 3,700 | 11 |
| | | Vertebra | 2,750 (1,400–8,500) | 11.2 (8.5–15) |
| Male 2 | February 2019 | Dental pulp | 0 | 0 |

*IQR, interquartile ranges

Figure S1. SDS-PAGE electrophoresis on 12% polyacrylamide gels of the two *Treponema pallidum* antigens used in this study. The left lane in each panel corresponds to the molecular weight marker.

A. Recombinant antigen: 2 different concentrations (8 µg and 4 µg).

B. Native antigen: 2 different concentrations (10 µg and 5 µg).

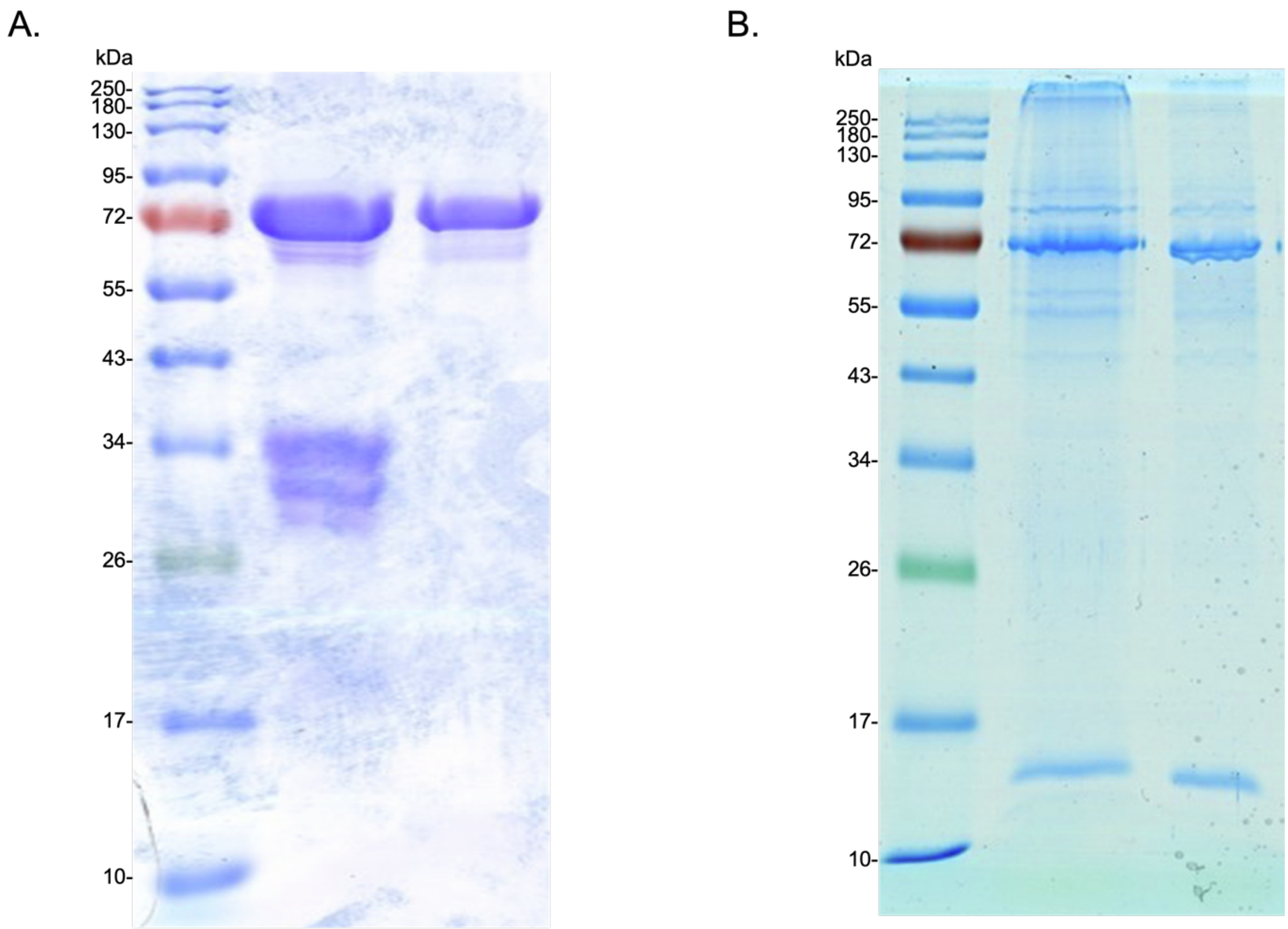

Supplement: Supplementary file 1 [file mmc1.pdf]
